# Mapping the conducting channels formed along extended defects in SrTiO_3_ by means of scanning near-field optical microscopy

**DOI:** 10.1038/s41598-020-74645-1

**Published:** 2020-10-20

**Authors:** Christian Rodenbücher, Karsten Bittkau, Gustav Bihlmayer, Dominik Wrana, Thomas Gensch, Carsten Korte, Franciszek Krok, Kristof Szot

**Affiliations:** 1 Institute of Energy and Climate Research (IEK-14), Forschungszentrum Jülich GmbH, 52425 Jülich, Germany; 2 Institute of Energy and Climate Research (IEK-5), Forschungszentrum Jülich GmbH, 52425 Jülich, Germany; 3 Peter Grünberg Institut (PGI-1) and JARA-FIT, Forschungszentrum Jülich GmbH, 52425 Jülich, Germany; 4 Marian Smoluchowski Institute of Physics, Jagiellonian University, 30-348 Krakow, Poland; 5 Institute of Biological Information Processing (IBI-1), Forschungszentrum Jülich GmbH, 52425 Jülich, Germany; 6 Institute of Physics, University of Silesia, 41-500 Chorzów, Poland; 7 aixACCT Systems GmbH, 52068 Aachen, Germany

**Keywords:** Electronic properties and materials, Surfaces, interfaces and thin films, Fuel cells

## Abstract

Mixed ionic-electronic-conducting perovskites such as SrTiO_3_ are promising materials to be employed in efficient energy conversion or information processing. These materials exhibit a self-doping effect related to the formation of oxygen vacancies and electronic charge carriers upon reduction. It has been found that dislocations play a prominent role in this self-doping process, serving as easy reduction sites, which result in the formation of conducting filaments along the dislocations. While this effect has been investigated in detail with theoretical calculations and direct observations using local-conductivity atomic force microscopy, the present work highlights the optical properties of dislocations in SrTiO_3_ single crystals. Using the change in optical absorption upon reduction as an indicator, two well-defined arrangements of dislocations, namely a bicrystal boundary and a slip band induced by mechanical deformation, are investigated by means of scanning near-field optical microscopy. In both cases, the regions with enhanced dislocation density can be clearly identified as regions with higher optical absorption. Assisted by ab initio calculations, confirming that the agglomeration of oxygen vacancies significantly change the local dielectric constants of the material, the results provide direct evidence that reduced dislocations can be classified as alien matter embedded in the SrTiO_3_ matrix.

## Introduction

Strontium titanate (SrTiO_3_) has become one of the most extensively studied metal oxides due to its exceptional electronic properties, which hold promising potential for applications in energy conversion and electronics. It has been demonstrated that SrTiO_3_ can be used for photocatalytic water-splitting^1^, as an anode in solid oxide fuel cells (SOFCs) or dye-sensitized solar cells^2,3^, as an oxygen sensor^4^, as a substrate for the generation of 2D electron gases^5^ or high-temperature superconductors^6^ and as memristive material^7^. A key feature of SrTiO_3_ is that its electronic transport properties are closely related to oxygen nonstoichiometry, which can be manipulated via redox reactions^8^. Upon reduction, oxygen is excorporated from the lattice, leaving behind two-fold positively-charged vacancies that are compensated by electrons. In consequence, a valence change in the transition metal ion Ti from + 4 to + 3 occurs^9^. In this way, the oxide can be turned from an initially insulating state into a highly conducting and ultimately metallic state^10^. In the first stage of reduction, the oxygen vacancies are not homogeneously generated within the crystal, but a preferential reduction in the extended defects, such as dislocations, occurs. This results in the evolution of a network of metallic nanofilaments along dislocations within an insulating matrix^11–13^. In a typical, commercially-available single crystal grown using the Verneuil method, two sources of dislocation exist. Firstly, the fast growth process under non-equilibrium conditions leads to the evolution of dislocations with a density of approximately 10^5^/cm^2^ and secondly, cutting and polishing results in an increase in the dislocation density in the near-surface region, which can be several orders of magnitude higher than in the bulk^14–16^. Through nanoscale investigations, it can be seen that those dislocations appear as either single dislocations or as bundles of dislocations, causing significant inhomogeneity in the material properties. However, from a macroscopic perspective, with respect to samples with millimetre dimensions, the dislocations can be regarded as being randomly distributed, and that the related inhomogeneity will level out. However, when the dislocations are forced into oriented arrangements, a significant impact on the local electronic properties is to be expected. The electronic properties of reduced dislocations have been experimentally-investigated by measurements employing local conductivity atomic force microscopy (LC-AFM) on thermally-reduced single crystals. A high degree of heterogeneity, as well as a local confinement of the conductivity of each individual filament on the nanoscale, has thereby been observed^8,13,17^. To correlate the position of dislocations with that of the conducting filaments, samples with linear agglomerations of dislocations have been investigated. Mechanically-deformed crystals, with defined slip bands generated by bending, and bi-crystalline boundaries produced by the hot-joining of two tilt-cut crystals have been characterized by LC-AFM^13,18^. In both cases, an agglomeration of dislocations at the boundary and slip band were confirmed by the etch pits technique. The LC-AFM investigations revealed that close to the dislocation-rich regions, a high concentration of conducting spots related to the evolution of filamentary conductance paths is present^13,18^. Hence, both examples indicate that the local electronic transport of regions with enhanced dislocation density is distinctly increased compared to the bulk.

In this paper, we investigate the change of the optical properties of SrTiO_3_ upon thermal annealing on the macro- and nanoscale. By qualitative optical inspection combined with quantitative photothermal deflection spectroscopy (PDS) we analyse the influence of oxidation and reduction on the macroscopic optical properties. In order to investigate the optical properties on the nanoscale close to dislocations we employ scanning near-field optical microscopy (SNOM), which is a versatile tool to investigate the properties of solid oxides in a nanometer scale resolution^19–21^. SNOM has also been recently applied to investigate the local properties of grain boundaries and conducting filaments associated with resistive switching effect in SrTiO_3_^22^. Unless LC-AFM, the SNOM technique is capable of detecting conductance paths and dopant profiles not only at the surface but also in deeper buried regions of the sample due to the use of the optical near-field^23,24^. Here, we focus on the same two examples of linearly-arranged dislocations that we have previously characterized by LC-AFM, namely a bicrystal boundary and slip band. We demonstrate that the dislocation-rich structures can be clearly resolved by SNOM whose signal indicates the presence of conducting paths. Assisted by ab initio simulations, we demonstrate that the local electronic and optical properties of reduced dislocations distinctly differ from those of the bulk, presenting evidence for intrinsic inhomogeneity in dislocation-rich SrTiO_3_ crystals.

## Results and discussion

**Figure 1:**
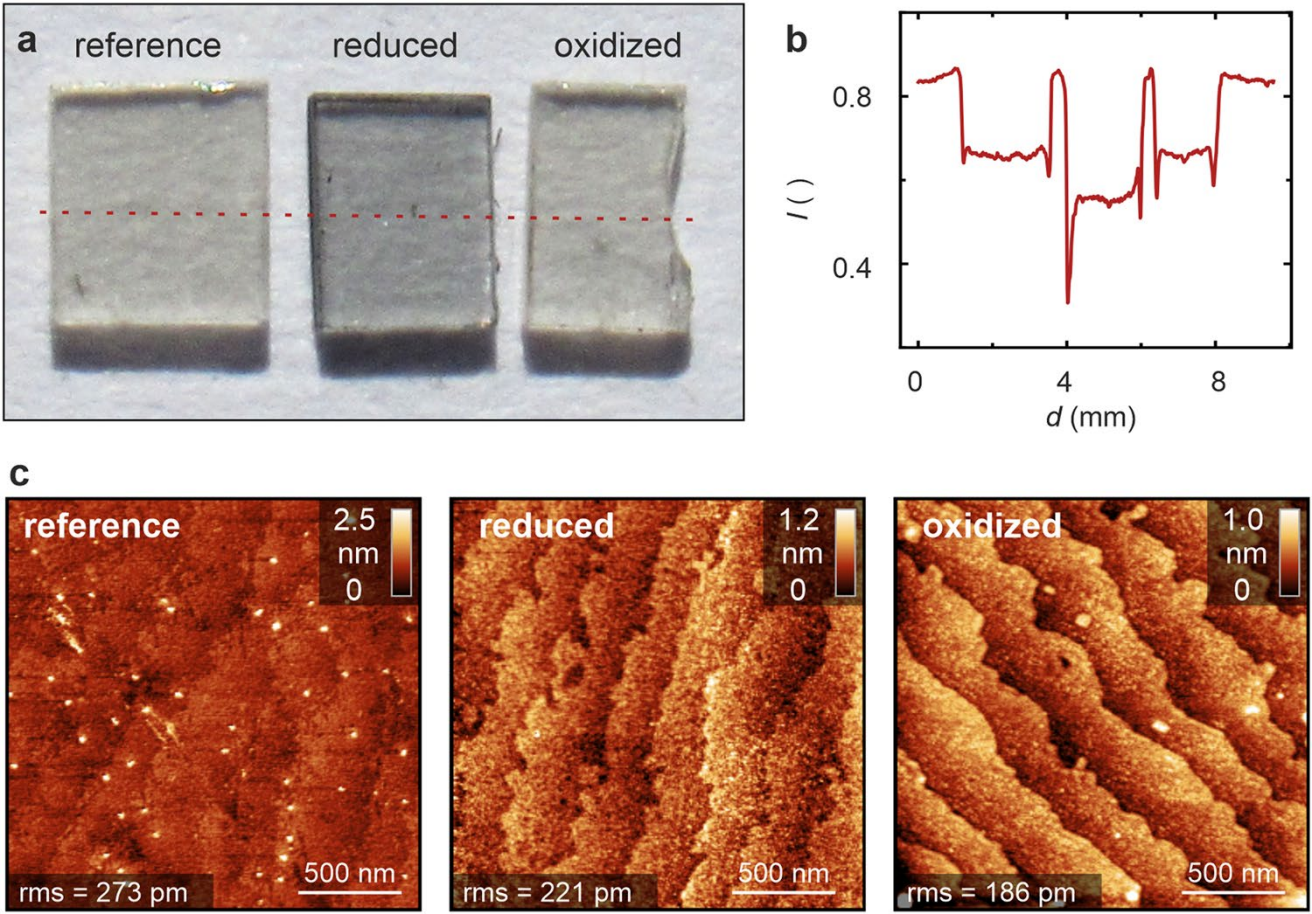
Influence of thermal annealing on the transparency and morphology of SrTiO_3_. (**a**) Optical micrographs of SrTiO_3_ single crystals before and after annealing under oxidizing and reducing atmosphere, (**b**) intensity value of the optical micrograph calculated along the red dashed line, (**c**) topography of the crystals measured by AFM.

First, we would like to briefly illustrate the influence of redox processes on the optical properties of SrTiO_3_. We compared an as-received reference crystal with a crystal that had been reduced under vacuum conditions at an estimated oxygen partial pressure of 10^–11^ mbar, and a crystal that had been oxidized under an oxygen partial pressure of 200 mbar. In both cases, the annealing temperature was 1000 °C and the annealing time was 1 h. After annealing, the samples were quenched down to room temperature within one minute. In Fig. 1a, the optical micrographs of the three samples are shown. The influence of the annealing conditions on the optical properties is visible even with the naked eye at the reference crystal, as well as at the oxidized crystal, being transparent, and the reduced crystal which had a slightly dark-bluish, translucent colour that corresponded to previous reports^25^. This observation reflects the well-known change in electronic structure when introducing oxygen vacancies, leading to self-doping, due to the increase in the electronic charge carriers^26^. In particular at the rims of the reduced crystal, the darkening was pronounced as exemplified by the cross section of the intensity *I* calculated as average of the RGB values (Fig. 1b). This appears to be related to the increased dislocation density at the rims produced by sawing which promotes the reduction locally^27^. To analyse the morphology of the crystals we employed atomic force microscopy as presented in Fig. 1c. While the surface of the reference sample was rather unstructured, as a result of the cutting and polishing procedure done by the manufacturer, on the surface of the samples annealed under reducing as well as under oxidizing atmosphere, defined terraces were present. This confirms the well-known effect that the exposure of the crystal to high temperatures leads to a surface restructuring^28^. Comparing the surfaces of the reduced and the oxidized sample, it reveals that the roughness of the reduced sample was higher than that of the oxidized one.

**Figure 2:**
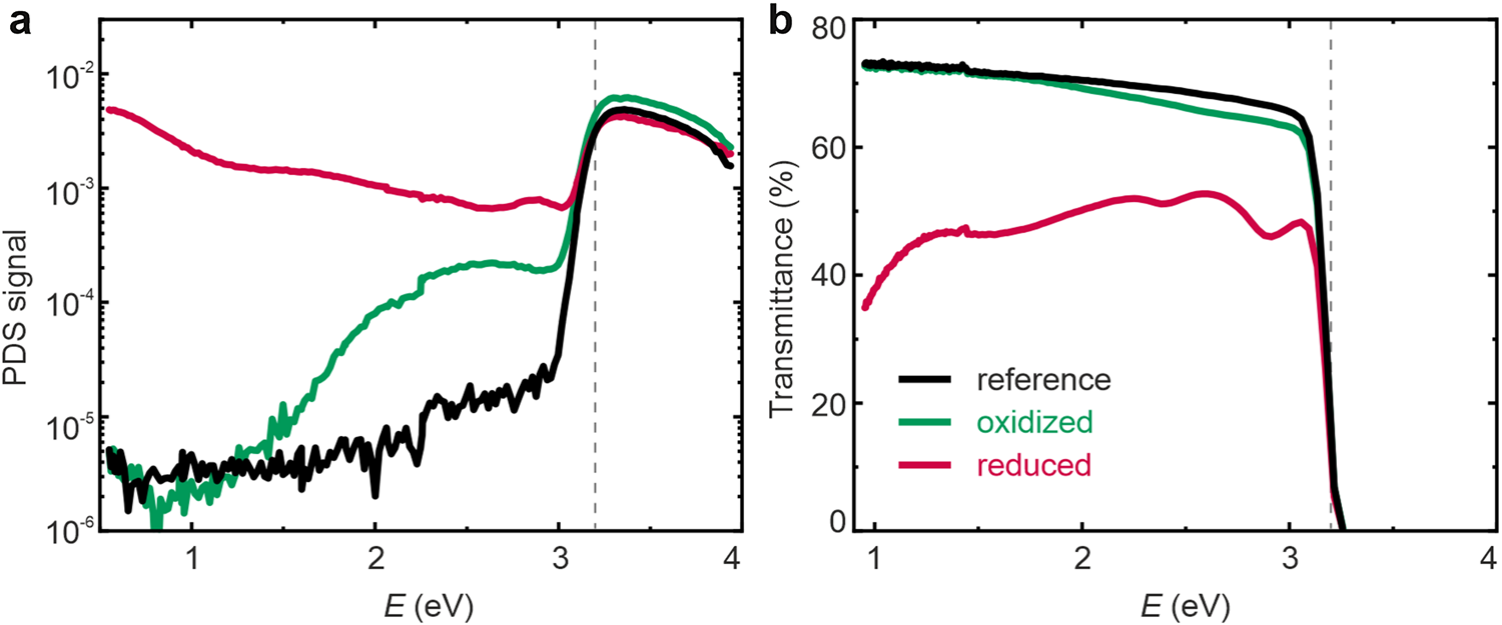
Optical analysis of the reference, oxidized and reduced SrTiO_3_ single crystals by PDS. (**a**) PDS signal, (**b**) transmittance. The dashed lines mark the band gap of 3.2 eV.

In order to quantify the changes in optical properties upon thermal annealing, we measured optical absorbance using the PDS technique. Figure 2 shows the PDS signal, which is proportional to the absorbance of the sample and the transmittance signal for the reference, oxidized and reduced crystal. In agreement with the literature, all three samples show full absorption above the band gap of approximately 3.2 eV^29,30^, but below this photon energy, significant differences between them can be identified. Here, the reference crystal shows the weakest absorption and is thus the most transparent to visible light. The absorption of the oxidized sample is slightly stronger than that of the reference sample in the region between 1.5 and 3 eV, which could possibly relate to the generation of Sr vacancies and the segregation of Sr to the surface^31^.

This effect has been confirmed using a variety of techniques including secondary ion mass spectrometry (SIMS) and X-ray photoelectron spectroscopy (XPS)^7,28,32^. During the oxidation process, the segregation of Sr to the surface can lead to a local formation of Ruddlesden-Popper phases in the surface layer and eventually to the evolution of SrO islands on the surface. As the samples in the present study were only annealed for 1 h, a distinct formation of islands on the surface was not observed (Fig. 1c). However, it can be expected that the Sr enrichment in the surface layer results in a change of the electronic and optical properties, detectable already in an early stage of the transformation. Indeed, blue colouring was observed for nonstoichiometric SrTiO_3_ ceramics with an Sr/Ti ratio greater than 1^33^. The reduced sample, however, shows significant absorption, resulting in a transmittance below 50% relating to the creation of vacancies and free charge carriers associated with the generation of Ti^3+^ ions^34^. Summarizing this macroscopic investigation, we can conclude that upon the creation of electronic charge carries at the point of reduction, the optical absorbance increases, resulting in a decrease in the transmittance.

**Figure 3:**
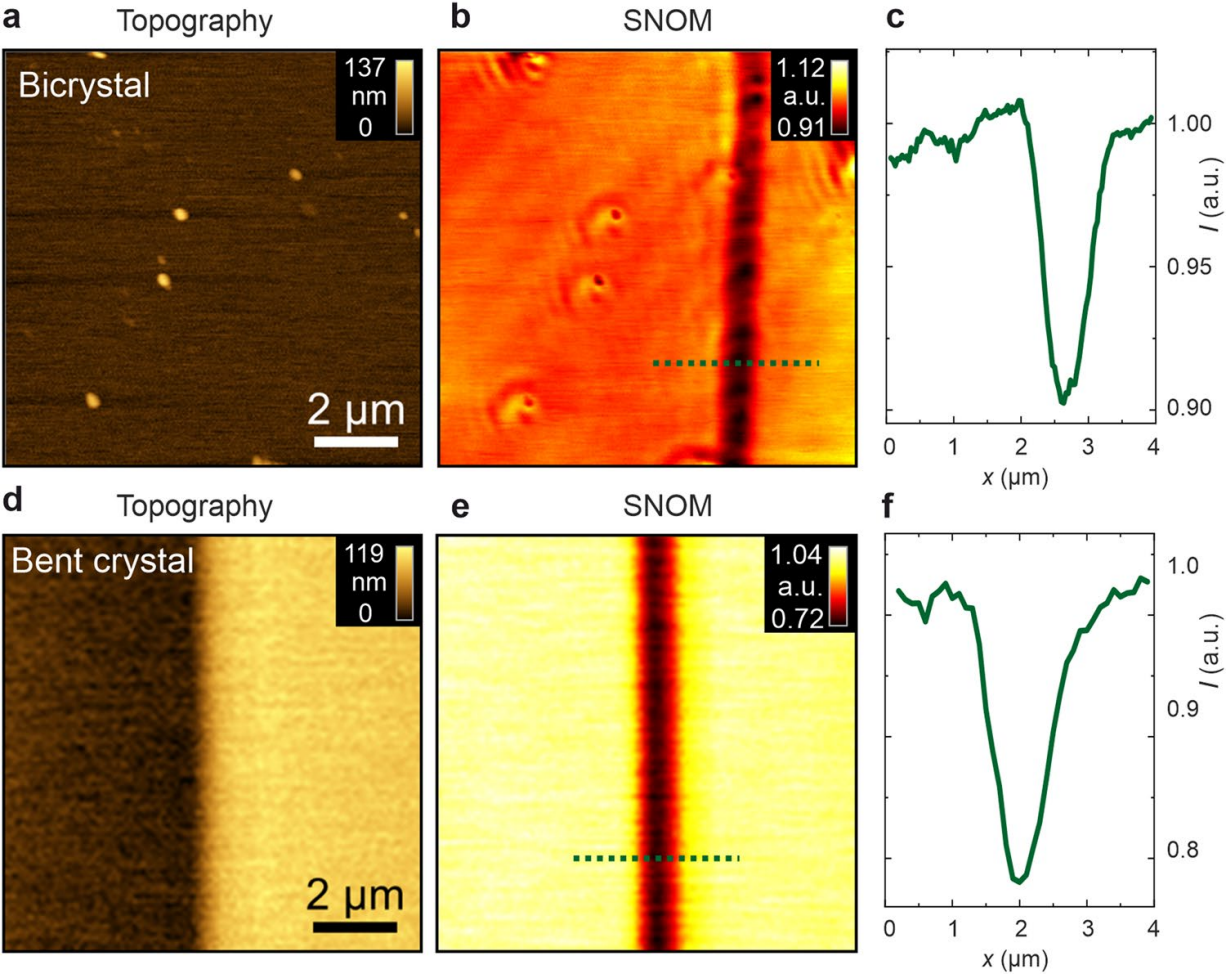
SNOM analysis of the dislocation-rich regions of SrTiO_3_. (**a**) Topography and (**b**) SNOM signal with (**c**) the line profile of a 36.8° bicrystal boundary. (**d**) Topography and (**e**) SNOM signal with (**f**) line profile of a slip band in a bent and reduced crystal.

Having verified the significant change in the macroscopic optical properties upon reduction, we will now focus on detecting the local modifications in optical transmittance on the nanoscale by means of SNOM measurements. In analogy to our previous LC-AFM investigations on the agglomeration of dislocations^13,18^, we investigate a bicrystal boundary and a slip band. In Fig. 3a–c, the investigation of the bicrystal boundary is shown. As the SNOM signal is measured by employing a tapered glass fibre tip via a quartz tuning fork, maps of the topography and transmittance can be measured simultaneously^35^. The illumination was achieved through the fibre tip and the transmitted light intensity was collected with a microscope objective and photomultiplier. In accordance with the previous LC-AFM results^18^, the topography at the boundary position is flat, as the bicrystal was epipolished after the junction of the two tilt-oriented crystals (Fig. 3a). Hence, no topography-related diffraction altering the SNOM signal is to be expected. In the SNOM intensity map measured using an incident light of 658 nm wavelength, the boundary can be clearly identified as a dark stripe with a transmission intensity reduced by 5–8% (Fig. 3b). The FWHM of the stripe is about 730 nm, which is similar to the width of the band of the conducting spots detected by LC-AFM^18^. As per the argumentation from the macroscopic measurements, we conclude that a higher concentration of charge carriers is concentrated at the dislocations of the boundary compared to the bulk. Furthermore, space charges forming around the cores of dislocations or the presence of different crystallographic phases may play a role in altering the local optical properties^36^. Along the dark stripe at the crystalline boundary, an aperiodic intensity modulation with an amplitude of about 2% is apparent. The lateral distance between two minima is in the range of 500–600 nm, which indicates that the dislocations are not regularly aligned at the boundary but that there is a certain degree of variation. Such variation has been attributed to deficiencies in bicrystal preparation by the hot-joining technique^13^. As a side effect, an additional structure can be observed in the SNOM transmission map at the position of the surface protrusions. Here, the intensity is locally reduced and accompanied by an increased intensity in the area surrounding the protrusions. This feature can be explained by light coupling efficiency through the near-field aperture, which depends on the interaction of the apex of the tip and the local surface variations^37^.

As a second case, we also analyzed the bent and reduced sample with mechanically-induced slip bands by means of SNOM (Fig. 3d–e). The steps induced by superplastic deformation on a SrTiO_3_ single crystal could be easily identified, indicating a sharp step with a height of about 100 nm in the topography map (Fig. 3d). To obtain the corresponding near-field transmission image, a wavelength of 488 nm was chosen to suppress the diffraction of incident light at the sharp step edge on the surface, which is present at longer wavelengths. We observed a stripe with reduced near-field transmission along the step and an intensity reduced by about 15% with a FWHM of about 1000 nm (Fig. 3f). The locally-reduced near-field transmission signal indicates that the boundary region absorbs more light than the rest of the crystal. Taking into account conclusions derived from the macroscopic PDS measurements, this indicates an increased charge carrier density along the agglomerated dislocations, which is to be expected, as the dislocations constitute preferential reduction sites.

**Figure 4:**
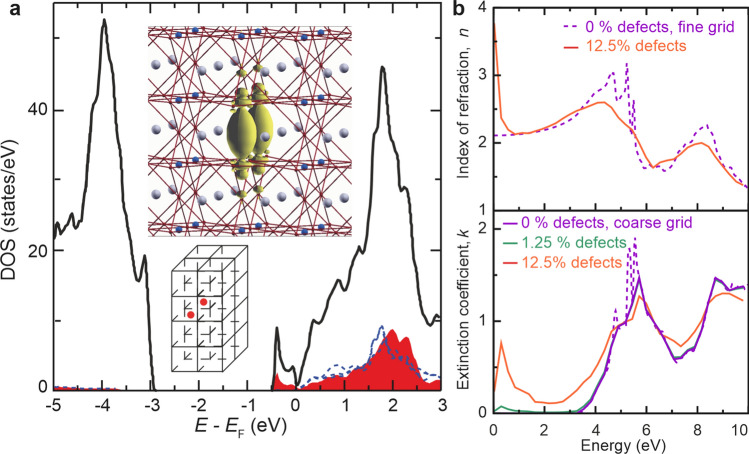
Ab initio simulation of an extended defect in SrTiO_3_. (**a**) The density of states (DOS) and location of the oxygen vacancies in the SrO planes of a 2 × 1 × 4 supercell of the crystal. The black line indicates the total DOS, while the red curve corresponds to the local DOS at the Ti positions above and below the defect. The blue line shows the LDOS at a Ti site far from the defect. The yellow iso-surfaces outline the charge density of the states between 0.5 eV and the Fermi level (E_F_). (**b**) Index of refraction and extinction coefficient of stoichiometric SrTiO_3_ (violet dashed lines) and SrTiO_2.875_ (orange line). In the lower panel, the result is further extrapolated to the situation with a ten-fold lower defect density (green line). Note that the calculations for the defective unit cell were performed on a coarser energy grid than the calculations without defects.

To gain closer insight into the electronic structure of reduced dislocations, ab initio simulations for stoichiometric SrTiO_3_ and a structure with an extended defect were performed on the basis of density functional theory using the full-potential linearized augmented plane-wave method as described by Al-Zubi et al.^38^. The real and imaginary parts, ε_1_ and ε_2_, of the inverse dielectric function, 1/ε_00_(**q**,ω), for **q** = 0 were calculated with the spex code^39^. The real and imaginary part of the refractive index, *n* and *k*, were obtained from the relation (*n* + i*k*)^2^ = ε_1_ + iε_2_. We chose a 2 × 1 × 4 supercell of SrTiO_3_ with an oxygen defect row in the SrO plane (Fig. 4a). This extended defect gives rise to a split-off band at the bottom of the conduction band with 0.5 eV bandwidth. Transitions from this band to the unoccupied Ti-*d* states precipitate a small peak in the extinction coefficient, *k*, at about 0.3 eV that falls off slowly, reaching a minimum at 2 eV. The tail of this peak appears at the same energy level where, in Fig. 2b, a drop of the transmission can be observed (between 1 and 2 eV). As the defect density in the chosen supercell is unrealistically high (12.5% oxygen vacancies), we make a linear interpolation between the dielectric function of defect-free SrTiO_3_ (violet lines) and the results obtained for high vacancy concentrations (Note that for this interpolation, both calculations had to be fitted to the same energy grid). The green line in Fig. 4b (ε_2_) corresponds to a defect concentration of 1.25%. Regarding investigations of local oxygen non-stoichiometry by high-resolution transmission electron microscopy (HR-TEM), one can safely assume that a relatively high vacancy concentration is present close to extended defects. This can be justified since our defect filaments do not interact strongly with each other. It has been shown that extended defects like edge dislocations locally adopt the structure of TiO_2-x_, Ti_2_O_3_ or TiO; i.e. of compounds with a very high concentration of oxygen vacancies^13^. It is important to note that the calculations show that absorption can be expected throughout the gap (with a slight increase towards lower energies) before the transmission drops to zero at 3.0 eV. This can be directly correlated to the SNOM measurements that were obtained with photon energies of 1.8 eV (658 nm) and 2.5 eV (488 nm) below the gap. The simulations predict that in this energy range, the transmission for a region with a high defect density is lower than that of a defect-free region. As the SNOM results indeed show a decreased transmission signal close to the bicrystal boundary, especially after reduction, the assumption that oxygen vacancies are agglomerated in the dislocation-rich area is supported.

## Conclusion

In summary, we have shown that the local optical properties of dislocation-rich areas in SrTiO_3_ differ from those in bulk. This effect has been attributed to an increased concentration of electronic charge carriers, leading to an increase in the optical absorption. This effect allows for the use of optical microscopy methods to map arrangements of dislocations such as those which form along a bicrystal boundary or in mechanically deformed crystals. The results are further evidence for the special role of dislocations as preferential reduction sites, as has been theoretically predicted. We demonstrated that the SNOM technique can be used as complementary method to measuring techniques requiring the direct contact to subject, such as the LC-AFM, as it can be performed with a high spatial resolution. This approach allows to conclude that dislocations, intrinsically present (e.g. in a bicrystal) or are artificially induced (e.g. by superplastic deformation), serve as a template for the evolution of filaments with a high concentration of oxygen vacancies compensated by electrons. We can assume that in this way, a network of conducting filaments is created within an insulating matrix upon the thermal reduction of SrTiO_3_. Hence, it must be taken into account that the electronic properties of reduced SrTiO_3_ single crystals can be highly inhomogeneous on the micro- and nanoscales, thus making the modelling of reduction-related effects such as electroforming, resistive switching or material degradation of mixed electronic-ionic-conducting oxides a demanding task. Similar localized effects have also been found close to LaAlO_3_/SrTiO_3_ interfaces, where a subtle interplay between conducting paths and the formation of ferroelastic domains below the cubic-to-tetragonal transition temperature is present^40–42^. Furthermore, we have shown that through mechanical deformation, conducting dislocation-rich regions in a defined manner can be induced. As optical transmittance has been found to be reduced close to such highly conducting regions, this would open up possibilities for the use of dislocation-rich SrTiO_3_ as quasi-transparent conductors, e.g., for electrodes in solar cells.

## Methods

Verneuil-grown, undoped SrTiO_3_ single crystals and bicrystals of 0.5 mm thickness with epi-polished surfaces in (100) orientation (Crystec, Berlin, Germany) were investigated. For the generation of slip bands, the crystal was manually deformed by bending around a supporting prism via superplastic deformation (for details of the procedure, see Szot et al.^13^ and Speier et al.^43^). Thermal reduction was performed in an evacuated quartz tube (*p* < 10^–6^ mbar) heated by a tube furnace. AFM measurements in intermittent contact mode were performed under ambient conditions using doped Si probes. Optical absorption was investigated by PDS measurements in a quartz cuvette filled with a non-absorbing deflection medium (here CCl_4_), as described by Jackson et al.^44^. SNOM measurements were obtained using an aperture-type setup providing sub-100 nm optical resolution, as described by Cao et al.^37^. Theoretical calculations of the dielectric constants of an oxygen-deficient extended defect in SrTiO_3_ were conducted using the spex code based on simulations using density functional theory (for details, see Al-Zubi et al.^38^ and Friedrich et al.^39^).

## Data Availability

The datasets generated during and/or analysed during the current study are available from the corresponding author on reasonable request.
